# Supplementary material for: Are carbon emissions trading and green financial instruments synergistic? -Comprehensive quantitative research based on content analysis
Source: PLoS One. 2024 Mar 7;19(3):e0298601. doi: 10.1371/journal.pone.0298601 (PMC10919601; doi:10.1371/journal.pone.0298601)
Supplement: S1 Data — (ZIP) [file pone.0298601.s001.zip › Data/地级市Z_F工作报告.zip!/─■╧─/╩»╫∞╔╜╩╨/└┤╫╘╣1⁄2╓┌║┼í╛┬φ┐╦╩2╛▌═°í┐_2014.docx]

各位代表：

现在，我代表市人民政府，向大会作政府工作报告，请予审议，并请政协委员和列席人员提出意见和建议。

2013年工作回顾

2013年，面对严峻复杂的宏观经济形势，市人民政府在自治区党委、政府和市委的正确领导下，在市人大和市政协的监督支持下，全力以赴稳增长、调结构、惠民生，推进产业、民生和生态转型，经济社会稳中有进、稳中提质。全市地区生产总值达到446亿元，增长10%；固定资产投资459亿元，增长20.5%；完成地方公共财政预算收入31亿元，同比增长13.2%；城镇居民人均可支配收入22224元，农民人均纯收入8928元，分别增长9.5%和12.1%；城镇登记失业率为3.51%；节能减排指标按自治区下达目标完成。

——有效应对下行压力，工业经济平稳增长。针对工业经济低迷的严峻形势，强化运行调控，及时出台电价补贴、扩大销售、贷款贴息等政策，全力落实工业稳增长和“双龙工程”扶持政策。克服财政增收困难，兑现扶持资金5.1亿元，减免退抵税收7.3亿元。继续实行市级领导和县区、部门负责人结对联系重点和困难企业责任制，协调解决企业项目审批、融资贷款、市场销售、恢复生产等问题。采取有效措施，逐步化解煤炭行业和部分实体企业信贷风险。围绕推进矿山机械装备产业基地建设、有色金属新材料产业基地建设、电石化工升级改造工程三个重大专项，狠抓优势特色产业培育和技改升级，组建冶金集团、钢铁集团，成立宁夏氰胺行业协会，新增规模以上企业30家。实施重点工业项目22个，完成投资64亿元；实施中色东方、福华冶金等技改项目188个，完成投资135亿元；申银特钢、丽珠医药等52个工业项目建成投产。实施园区循环化改造项目10个，启动高载能企业搬迁改造，完成15家电石、铁合金企业炉型置换37台、109万千瓦。投资15亿元，加强四个工业园区水、电、路等基础设施建设。开发区扩区调位稳步推进，出台了加快高新技术产业园区发展的意见，下放经济技术开发区24项市级行政和经济管理权限，高新技术产业园升级为国家级高新区，生态经济开发区升级为自治区级经济技术开发区。全市规模以上工业实现增加值200亿元，增长12.1%。

——倾力推进项目建设，发展后劲不断增强。在实行市级领导包推重点项目责任制基础上，完善“六个一”工作机制，组织三批重点项目集中开工和两次全市项目建设现场观摩点评会，111个重点项目完成投资220亿元。德信恒通、中电科光伏发电、国际建材城、生态经济开发区路网、舍予三产区基础设施配套等19个项目顺利竣工；大地轮胎、力帆凯马汽车、吉元冶金、日盛化工、综合商贸城、西部吉运物流园、恒产总部商务区、棚户区改造等35个项目快速推进。

开展“十二五”规划中期评估，完善了重点项目、产业结构调整、“双龙工程”和试验区建设四个项目库，共储备项目339个，计划总投资4263亿元。抢抓内陆开放型经济试验区建设和全国老工业基地调整改造机遇，强化争项目争资金力度，争取国家、自治区各类资金69.6亿元，同比增长17.2%。修订完善了招商引资优惠政策，主动对接中东部省区，加强与央企的联系，引进招商项目288个，实际到位资金359.8亿元，同比增长21%，招商引资在逆境中实现突破。

——统筹推进区域建设，城乡一体协调发展。现代农业稳步发展，粮食连续10年增产增收。东方羊毛、超娃米业、沙湖清真食品等农副产品加工企业迅速成长，清真牛羊肉、制种、水产等优势产业规模效益不断提高，农产品加工转化率达到57%，被科技部确定为国家农业科技园区。实施了永兴生态养殖园、昊丰种业、全泰兴农生态养殖、高仁清真牛羊肉产业园等122个村镇招商项目，完成投资13.7亿元。启动银北百万亩盐碱地改良工程，土地开发整理、高标准基本农田建设及汝箕沟防洪二期工程、第五排水沟综合整治等项目顺利推进，完成中低产田改造10.2万亩，新增节水灌溉面积12万亩，全面完成耕地保护目标任务。培育家庭农场53家，新增土地流转面积6.2万亩，被确定为“全区农村经营机制创新试点市”。土地集约节约利用水平显著提高，大武口区被评为全国土地集约节约模范区。积极探索“三权抵押”贷款模式，办理贷款910笔5300多万元，在农村土地产权抵押贷款上实现零的突破，平罗农村土地经营管理制度改革试验区顺利通过农业部中期评估。实施旧村整治25个、改造农村危房2285户，礼和乡、崇岗镇2个重点小城镇和兴民村、聚宝新村等10个幸福村庄建设加快。石嘴山新火车站、平罗汽车客运站投入运营，简滨公路(西段)、红崖子至陶乐公路等工程顺利推进。

——持续加强生态建设，人居环境显著改善。以创建全国文明城市为龙头，推进“六城联创”，深入开展公共卫生、环境整治等攻坚战，成功创建国家森林城市。平罗被评为自治区文明县城、卫生县城。实施百万花灌木进城、星海湖中域绿化和南域整治、移民安置区绿化等35个重点生态项目，完成植树造林10万亩。组织实施了大武口城市雨污分流管网改造、惠农老城区街路改造等工程，新建、改造停车场18个，建设特色街区5条，完成18条城市街路亮化改造、14条城市道路硬化工程，改造老旧小区22万平方米，城市环境和形象进一步提升。全力推进主干道路大整治大绿化，累计投入5.8亿元，拆除违章建筑、危旧房屋117万平方米、大型广告牌76块、清理垃圾96万吨。规范发展煤炭市场，累计清理取缔非法经营户515家，建成储煤仓286个，三个煤炭经营集中区全部实现封闭运行管理。加大污染治理力度，实施完成39个减排项目，关停22家不符合产业政策的企业，淘汰落后产能298.7万吨。深入开展工业园区、矿山地质环境、砂石资源等综合整治，生态环境进一步改善。

——加快实施十大专项，内陆开放取得进展。围绕“四大战略定位”，在扩大开放合作、体制机制改革、优化政策环境等方面重点突破，实施10个重大专项，成为试验区建设有力抓手。以惠农陆港口岸列入自治区试验区建设“8+1”方案为契机，积极协调推进宁夏与天津两区市高层对接。“智慧石嘴山”建设稳步推进，顶层设计顺利完成，电子政务信息平台调试运行，我市被列为国家智慧城市试点。特色化工品基地获批国家外贸转型升级示范基地。石嘴山黄河大桥开工建设，红赛公路建成通车，周边物流通道进一步畅通。星海银河景区、景观绿化及水域治理等环湖开发项目加快推进，星海湖被评为中国文化旅游新地标，沙湖跻身全国十大魅力湿地，我市被列入“中国文化旅游示范基地”。整合职业教育资源，组建石嘴山联合学院，宁夏旅游学校与我市合作办学。进一步完善全国开发区合作发展交流机制，与东部4个开发区建立友好合作关系。加强与阿拉伯国家和穆斯林地区交流合作，与约旦亚喀巴经济特区、法国帕亥勒蒙尼亚市缔结为友好城市。与清华、北大签订人才合作协议，34名清华大学研究生来我市开展社会实践和挂职锻炼。积极创新融资方式，加快推进直接融资，日晶新能源、天润新能源和绿恒科技在天交所挂牌上市，石嘴山银行发行5亿元金融债券，晟晏福华6.4亿元企业债、4亿元短期融资券通过审核择期发行，星瀚集团8亿元城投债上报国家发改委待批。大武口农村信用联社改制为石嘴山农村商业银行。

——优先保障民生投入，社会事业稳步发展。克服财政增收困难，确保民生支出，市级用于民生事业投入达42.1亿元，占公共财政预算支出的73.2%，为民承诺的10件民生实事顺利完成。教育体育事业快速发展，荣获“全国最佳教育公平示范城市”称号。成功举办全国铁人三项锦标赛、室内五人制足球赛和全国露营大会。文化惠民工程深入推进，科技馆、博物馆免费开放，被列入国家公共文化服务体系示范区创建城市。创业促就业成效显著，创建孵化基地和创业园41个，发放再就业小额担保贷款3.49亿元，被列为全国职业培训示范城市创建和百家职业技能振兴活动城市。社会保险体系进一步完善，在全区率先试行病残津贴和城乡居民大病医疗保险制度，实现城乡居民基础养老金和城乡居民医疗费用报销比例双提高。强化价格调控和监督检查，居民消费品价格指数控制在3.3%以内。加快棚户区改造，建成棚户区安置房7504套60万平方米，廉租房、公租房、经济适用住房2894套15.4万平方米，10398户住房困难群众喜迁新居。全力做好移民工作，建成移民安置房3197套，搬迁安置4296户。创新安置方式，实施移民插花安置，移民安置区就业、社保、教育、卫生、社会治理工作扎实推进。街道(乡镇)“两个中心”规范运行，城乡社区网格化管理不断加强。深入推进“平安石嘴山”建设，严厉打击各类违法犯罪活动，积极化解社会矛盾纠纷和信访突出问题，维护了社会和谐稳定。高度重视安全生产，率先在全区推行应用企业隐患自查自报体系。人口计生、妇女儿童、老龄及残疾人保障、民族宗教、拥军优属、防震减灾、市场监管、食品药品、司法行政、国土资源管理、国企改革、统计调查、档案供销等工作都取得了新成绩。

——着力加强自身建设，行政效能不断提升。修订完善了政府工作规则和常务会议规则，建立并实行人大代表、政协委员列席政府常务会议制度。成立了政府顾问团、法律顾问团和经济运行咨询委员会，促进政府决策科学化、民主化和规范化。成立8个重大专项督查组，加大重大决策、重点项目监督检查通报力度，推动了工作落实和提质增效。强化审计监督，追缴财政资金1亿元。自觉接受人大法律监督、工作监督和政协民主监督，主动加强沟通联系，联合开展重点工作调研，认真办理人大代表议案、意见建议69件，政协委员提案209件，办复率为100%。全年办理各类网帖1590条，反馈率98.4%。坚决贯彻落实“改进工作作风、密切联系群众”的各项规定，出台控制行政事业单位运行成本十项措施，腾退办公用房1.1万平方米，“三公”经费支出同比下降13.56%。取消调整行政审批事项127项，减少审批环节257个。具有行政审批职能的单位集中进驻政务大厅，高效办理行政审批和服务事项，禁止向办事人员收取复印费、停车费等便民措施，得到社会各界好评，《人民日报》等多家媒体予以宣传报道。

在肯定成绩的同时，我们清醒地认识到，在发展中还存在一些不容忽视的矛盾和问题。主要表现在：经济结构不合理、产业发展层次低，结构调整和转型发展任重道远；工业企业大多处于产业链低端，产品附加值低，抵御市场风险能力弱，受市场波动影响大；支撑转型发展的大项目不多，缺少国家、自治区层面的重大项目，经济发展后劲不足；由于财政增收乏力，民生投入的不可持续性问题日益突出；个别部门和干部执行不力、效率不高、作风不实、工作落实不到位，缺乏有为和担当意识，行政效能有待提高，政务环境仍需优化，有些体制机制还不适应转型发展、科学发展的要求等等。对于这些问题，我们将在今后的工作中认真加以解决。

各位代表，回顾一年来的工作，我们经历了宏观调控新变化，经受了复杂局面新考验，成绩来之不易，更需倍加珍惜。这些成绩的取得是自治区党委、政府和市委科学决策、坚强领导的结果，是各位人大代表、政协委员有效监督、倾力支持的结果，是全市广大干部群众团结一心、攻坚克难的结果。在此，我代表市人民政府，向一年来辛勤工作在各条战线，为全市经济社会发展做出积极贡献的广大干部群众，向驻石部队、武警官兵、公安干警，向各民主党派、工商联、各人民团体和社会各界人士致以崇高的敬意!向关心支持石嘴山发展的中央、区属驻石各单位和各界朋友表示衷心的感谢!

2014年主要任务

今年是深入贯彻落实党的十八届三中全会精神，全面深化改革的第一年，也是完成“十二五”规划目标的关键之年，政府工作的总体要求是：全面贯彻落实党的十八大及十八届二中、三中全会，自治区第十一次党代会及自治区党委十一届三次全委会，市第九次党代会及市委九届四次全委会精神，围绕主题主线，坚持稳中求进，抢抓内陆开放型经济试验区建设、老工业基地调整改造和实施资源型城市可持续发展规划重大机遇，进一步深化改革、扩大开放，坚定不移推进产业、民生、生态转型发展，为建设开放富裕和谐美丽新型工业城市、在全区率先全面建成小康社会奠定更加坚实的基础。

主要奋斗目标是：地区生产总值增长10%左右；全社会固定资产投资增长20%左右；地方公共财政预算收入同比增长12%；城镇居民人均可支配收入和农民人均纯收入分别增长11%、12%；城镇登记失业率控制在4.5%以内；完成自治区下达的节能减排指标。

以上目标的安排，既是实现“十二五”发展规划的客观需要，也是有效应对压力、加快调整转型的必然选择，更是我市在全区率先全面建成小康社会的内在要求。总体来看，今年我市经济社会发展面临的有利方面较多。党的十八届三中全会作出全面深化改革的总体部署，将充分释放各方面的发展活力，为我们转型发展创造了新机遇；宁夏内陆开放型经济试验区建设的深入推进，自治区产业转型升级和结构调整方案的有效落实，为我们转型发展增添了新动力；我市制定实施的推进内陆开放、产业结构调整方案、老工业基地调整改造和资源型城市可持续发展规划等，为我们转型发展明确了新举措。我们的发展方向更加明确、发展思路更加清晰、发展动力更加强劲，为新的一年取得更大进步、实现新的突破奠定了基础。同时，我们也要看到，国内外经济环境仍存在较大的不确定性，虽然全球经济已迈过拐点缓慢复苏，但经济下行带来的影响依然会持续较长一段时间，“宏观偏紧、微观困难”的总体形势将给今年工作带来严峻挑战和重大考验，保持经济社会持续健康发展的压力将会更大。我们必须进一步认识和把握今年的困难和机遇，稳中求进，改革创新；必须进一步增强紧迫感和责任感，迎难而上，只争朝夕；必须进一步坚定发展的信心和决心，勇于担当，攻坚突破，促进经济持续健康发展、社会和谐稳定。

一、调整优化结构，推动产业转型升级，在提高质量效益上攻坚突破

坚持以改革创新促发展，发挥市场对资源配置的决定性作用，不断完善产业转型升级推进机制。制定和实施好《石嘴山市产业转型升级和结构调整工作方案》，全力打好产业结构调整攻坚战，努力提高经济发展质量和效益，打造石嘴山经济升级版。

加快工业优化升级。紧紧抓住“石嘴山资源枯竭型城市转型示范区”被列入自治区产业转型升级和结构调整十大任务的机遇，围绕新材料、装备制造、冶金、电石化工四大产业集群，深入实施“双龙工程”，扎实推进工业三个重大专项，做优存量，做大增量，以优质增量调存量，力争规模以上工业增加值增长12%。把技术改造和创新驱动作为传统产业优化升级的重要抓手，探索组建有色金属新材料产业创新联盟，在核心技术、关键领域合力攻关，共享创新成果。重点抓好中色东方、维尔铸造、英力特化工等194个技改项目，加快12个园区循环化改造项目建设，完成技改投资135亿元；实施好30个重点工业项目，开工建设国电大武口2×350MW热电联产、墨星新能源煤焦油加氢、科捷锂电池、华仪风电等项目；加快推进大地轮胎、力帆凯马汽车、金晶玻璃等项目建设，完成投资117亿元。对现有企业按竞争力分类施策，鼓励有实力的企业通过兼并重组、产业整合、上市融资做优做强，支持中色东方、大地化工、申银特钢向百亿元企业迈进。积极扶持“专、精、特、新”中小微企业发展壮大，形成大中小配套、多集群支撑的工业发展新格局。抓好经济技术开发区、高新技术开发区、生态经济开发区、宁夏精细化工基地扩区调位，完善基础设施，全力推动淄山工业园、科技创业孵化园等专业园区建设，争取生态经济开发区列入全国园区循环化改造试点。加强工业经济运行协调，落实扶持政策措施，帮助企业解决发展难题。高度重视实体企业金融风险防范，实现工业经济稳定安全增长。

提升服务业发展水平。坚持突出重点，增量发展，扩张提升陆港带动的生产性服务业。启动电子口岸建设，不断完善陆港口岸综合服务功能。深化与天津市合作，推动两地合作协议落实，加快实施宁津物流园项目。大力发展物流业，力促西部吉运物流园、申银物流园、综合商贸城等建成运营，繁荣发展国际建材城、万博汽车修配城等专业市场，启动平罗火车站周边改造。以扩大和吸引消费为目标，推进红星美凯龙、万德隆商务广场、万宇小商品美食文化广场、宏泰商业广场等城市商贸综合体建设，培育新的消费增长点。推进快递服务业与电子商务联动发展，拓宽消费渠道。按照“观景沙湖、休闲星海”理念，大力发展沙湖带动、环星海湖综合开发支撑的文化旅游业。全力推进与柳州东化、中基浩泰等企业合作项目，完善环星海湖区域基础设施，实施环湖景观绿化工程，加快水系治理优化，抓好奇石文化城、星海银河景区、澜波湾、星海湖葡萄酒国际度假城等项目建设。注重旅游与文化融合，提速五七干校文化园、北武当生态旅游区等项目，推进星海湖国家文化产业试验园区建设。以星海湖为平台，引入市场机制，积极争取并办好各类赛事及文化活动。加快大武口南区医院、学校、商贸等公共服务设施配套，多措并举解决建成区商品房销售问题，有效降低空置率。鼓励引导房地产企业向工业地产、文化旅游地产和棚户区改造等领域拓展，促进房地产业健康发展。力争第三产业增加值同比增长9%。

大力发展现代农业。启动国家农业科技园区建设，高标准推进农产品精深加工基地，发挥“一园四区”和8个现代农业科技示范园的引领作用，做大做强中粮米业、屯盛蔬菜、万嘉羊业、长湖清真产业园等农产品加工龙头企业，推动制种、清真牛羊肉、水产、蔬菜等产业区域化布局、规模化发展，全市制种面积达到13.5万亩，牛羊饲养量达到300万只(头)。

积极推广太阳能温棚新模式，加快中节能光伏大棚项目建设。加快构建新型农业经营体系，积极培育新型职业农民，年内培育家庭农场50家，专业大户1000户，全市土地流转面积达到50万亩。新培育农村专业合作经济组织20个，带动农民4万户。推行农业种养新技术，实施良种选育和推广工程，重点抓好高产、优质、高效水稻新品种示范推广，强化精耕细作和田间管理，扩大优质水稻种植规模。加快农资供应网络和经营流通体系建设。加强耕地保护，把百万亩盐碱地改良作为提高农业综合生产能力的一号工程强力推进，抓好灌区续建配套、大型沟道整治等项目实施，完成高标准基本农田建设10万亩，改造中低产田15万亩，新增节水灌溉面积12万亩。

发展内陆开放型经济。围绕建设开放石嘴山，搭建对外合作交流平台，拓展对外开放空间，提高内陆开放型经济发展水平。完善市级领导包抓、牵头单位负责、协作单位配合的重大专项推进机制，实施试验区29个重点项目，力促十个重大专项建设取得明显进展。强化与周边地市在资源、产业等领域的开发合作，主动融入呼包银榆、丝绸之路经济带，推进跨区域产业协作和重大基础设施建设。完善陆港口岸运营机制，实现一次申报、一次查验、一次放行，扩大“区域通关”和“直通放行”适用企业范围。畅通周边物流通道，加快石嘴山黄河大桥建设，争取红礼黄河大桥、东乌铁路连接线项目。积极融入中阿博览会，筹办中阿友好城市合作论坛，加强与约旦亚喀巴经济特区等友好城市的交流合作，争取与印度尼西亚、马来西亚签订2个友好城市协议。引导和鼓励企业、商会、行业协会开展对外经贸、教育、文化、科技交流活动，拓宽发展平台。

二、坚持以人为本，努力办好民生实事，在增进民生幸福上攻坚突破

按照守住底线、突出重点、完善制度的思路，把更多的财力、物力投入到惠民富民上，努力为社会提供多样化服务，更好满足群众需求，实现发展成果更多更公平惠及全市人民。

推动社会事业进步。加快教育事业发展，优化学校布局，深入推进“两名三提高”工程，加快智慧校园“校校通”、“班班通”项目建设，加强高中阶段教育国际合作交流，探索举办“国际课程班”，力争在全区率先实现义务教育发展基本均衡市。努力办好重大体育赛事。着力改善基层医疗卫生机构基础条件，新建市妇幼保健院和儿童医院，加快第一人民医院内科综合楼、第三人民医院改扩建等项目建设。更加注重困难群体、弱势群体和残疾人生活改善，推进“9073”养老模式，启动建设惠农龙辰苑老年公寓项目，加快建设永峰养老院。

完善社会保障体系。加快推进社会保障体系建设，抓好零就业家庭和移民参加社会保险，推进社会保障“一卡通”工程。加强企业劳动保障守法诚信建设，完善行政执法与刑事司法联动机制，切实维护劳动者权益。深入推进全国家政服务试点城市建设，实现城市社区家政便民服务网络全覆盖。着力促进就业创业，加大政府购买公共服务和公益性岗位力度，全年新增城镇就业1.8万人。加强移民技能培训和就业指导，积极搭建移民就业创业平台，争取星海镇列为自治区级移民就业示范基地。

繁荣发展文化事业。实施文化惠民工程，推进国家公共文化服务体系示范区创建，实施市图书馆数字化系统改造，抓好市非物质文化遗产保护中心、乡镇(街道)文化站、社区电子阅览室等项目建设，争取西部标准30项61个指标中70%的任务达标。拓展文化产业投融资渠道，培育一批文化骨干企业。加大文艺人才培养力度，继续办好全国露营大会、园林奇石节等特色文化品牌活动，创作一批具有地域特色的文艺精品，增强石嘴山文化影响力。

提高社会治理水平。规范街道(乡镇)“两个中心”运行，实行“网格化”管理，按照人性化和精细化的要求，向居民提供多元化服务。推行“四位一体”物业管理模式，提升物业管理水平。切实加强移民区社会治理，积极推行”5531″管理模式，引导移民逐步改变生产生活方式。开展矛盾纠纷排查化解工作，完善社会稳定风险评估机制，健全人民调解、行政调解和司法调解联动工作体系，开展网上信访受理，认真做好信访积案化解工作。依法管理民族宗教事务。做好流动人口、特殊人群管理服务，加强食品药品安全、交通运输等重点行业、重点领域监管，健全防灾、减灾、救灾体系，提高突发事件应急处置能力。强化安全生产监管，全面落实企业安全生产主体责任，坚决遏制重特大事故的发生，不断推进公共安全体系和平安石嘴山建设。

提速棚户区改造。按照“两年基本完成、三年彻底完成”的目标，全力打好棚户区改造攻坚战，棚户区改造续建10564套85万平方米，新建8955套72万平方米，完成5000套40万平方米。严格落实责任追究，着力解决好房屋征收、资金筹集等突出问题，加快医院、学校、商贸等公共服务设施配建。通过置换或购买现有商品房，扩大棚户区安置房源。实行最严格的质量管理，切实把民心工程建成“放心工程”。

今年，市政府将认真办好十件民生实事。1.加快棚户区改造和公租房、廉租房建设，完成6000套45万平方米建设任务；2.实施20万平方米老旧住宅小区节能改造；3.改造10条城市街路人行步道，硬化15条居民区巷道，新改建20个城市免费公共卫生间；4.为全市一级重度贫困精神残疾人发放住院补助，为困难群众免费办理法律援助诉讼案件800件；5.实施智慧校园暨“班班通”项目，建设10个校园网络、300个“班班通”；6.建设市妇幼保健院和儿童医院，增强妇女儿童医疗保健服务能力；7.实施文化惠民工程，建设30个社区文化活动中心公共电子阅览室；8.实施大气污染防治，建设3个工业固废处置场；9.完成移民职业技能培训1800人，培训后实现70%稳定就业，扶持100名移民实现自主创业；10.建设港湾式公交停靠站70处，延伸完善城市公交线路。

三、加强环境保护，促进资源节约利用，在生态文明建设上攻坚突破

坚持生态立市和可持续发展，不断完善环境治理和生态修复机制，实行最严格的源头保护制度，进一步提升生态文明建设水平。

大力开展生态建设。以高速公路城市过境段、国省干道为重点，着力打造主干道路沿线绿化精品，完成主干道路两侧绿化350公里，绿化面积2万亩，推动大整治大绿化深入开展。巩固提升国家森林城市创建成果，建立造林绿化和林木管护长效机制。在每个县区规划筹建一个1000-1500亩以上的市民休闲森林公园，全年完成植树造林8万亩。推进矿山环境治理，进一步完善贺兰山东麓防洪体系建设，实施大武口归韭沟景观水道、惠农柳条沟治理等山洪、河洪治理工程。加强水资源保护，合理开采地下水。

规范发展煤炭市场。按照“巩固、提升、补欠”的要求，集中攻坚，进一步规范发展煤炭市场，全面完成“三年计划”目标任务。继续清理取缔非法煤炭经营户，规范煤炭集中区运行管理，推进储煤仓建设和使用，完善水、电、路等基础设施配套，加强集中区环境整治，初步实现市场秩序良好、环境明显改善的目标。建立完善长效机制，巩固扩大煤炭市场规范发展成果。

推进大气污染防治。以实行新环境空气质量标准为契机，实施大气污染防治行动计划，推进环境空气质量监测体系建设和大气污染防治工程。加大治水、治气、治渣力度，加强工业园区、城乡接合部等重点区域、重点污染源综合治理。对电石、铁合金等重点污染源实施24小时在线监测。严格工业园区固体废弃物管理，建设固废无害化处置场，实行集中堆存，覆土绿化。加大路面、建设工地扬尘和噪音污染治理力度。规范矿产资源开发，开展煤炭、砂石料等行业专项治理，严厉打击偷盗采行为。

强力推进节能减排。鼓励支持企业实施炉型置换、烟气余热利用、能量系统优化等节能示范工程，按照“总量控制、以大置小”的原则，完成12家冶金、电石企业搬迁改造、炉型置换和5家企业烟气余热综合利用。严格控制电石、铁合金等高载能企业产能总量，建立淘汰落后产能企业名单公告制度，落实限制类和淘汰类企业差别电价政策，淘汰和关停铁合金、电石、碳化硅三个行业落后产能38万吨。坚决落实节能减排目标责任制，确保年内单位GDP综合能耗下降2.76%。

四、突出规划引领，加快城乡一体步伐，在推进新型城镇化上攻坚突破

以新型城镇化为统领，优化空间布局，提升城镇质量，引导农民向城镇集中，推进农业转移人口市民化，加快城乡一体化进程，形成城市、城镇、乡村合理布局、功能互补、协同发展的格局。

完善城乡规划体系。按照《宁夏空间发展战略规划》，对现有的城镇总体规划、专项规划和重点区域规划进行修编完善，科学定位各县区城镇空间布局和区域功能，切实发挥规划对城镇建设的引领作用。完成《全市村镇布局规划》，实现乡镇和村庄建设规划全覆盖。加大规划执法力度，切实维护规划的严肃性、科学性和连续性。

提升城镇建设水平。加快推进大武口城市功能扩容提质、惠农城区街路改造、平罗旧城改造等项目建设，不断完善城市道路、管网等基础设施，提升城市公共服务功能。推进同城化路网建设，完成简滨公路、玉皇阁大道西延伸段、石嘴山至银川公路等工程，扎实做好京藏高速公路改扩建等项目前期工作。积极稳妥推进城市公交线路改造，提高公交覆盖面，方便群众出行。扎实推进智慧城市建设，完善基础数据库，建成市、县、乡、村四级信息网络体系，推进智慧城管、智慧交通和园区、企业信息平台建设，建成4G宽带网络并投入运营。

开展美丽乡村建设。抓好2个美丽乡镇和10个美丽村庄创建工作。充分发挥中心城镇的辐射带动功能，加快乡镇产业培育，支持发展具有乡村特色的休闲旅游、观光农业及特色餐饮等产业。实施农村环境卫生综合整治，做好农村污水处理和垃圾收集转运，基本完成农村土坯危房拆除。配套完善卫生医疗、购物网点等基础设施。以乡镇道路建设、村庄绿化、公共服务完善为重点，统筹推进，加大投入，努力构建充满活力、独具特色、环境整洁、风尚良好的宜居乡镇。

冲刺全国文明城市创建。深入实施素质提升工程，深化市民思想道德建设，规范市民文明行为，提高市民道德意识。实施城市街路亮化、道路硬化和老旧小区改造。开展城乡接合部、城区道路两侧和背街小巷综合整治，完成城市广告牌匾整治、环卫设施改造，加快特色街区创建，不断改善城市形象。以创建全国文明城市为龙头，带动国家园林城市、卫生城市、法治城市和全国“双拥”模范城市创建，全面提升城市文明程度。统筹抓好创城基础工作，确保重点工作、硬件建设和台账任务全部达标，全力跻身全国文明城市行列。

各位代表，做好今年的政府工作，责任重大，任务艰巨，我们必须以更加饱满的热情，更加务实的作风，更加有力的措施，突出重点，强力推进。

(一)抓项目建设，激发内生增长动力。坚持企业投资的主体地位，发挥投资的关键作用，始终把项目建设作为经济工作的总抓手，全力打好项目建设攻坚战，增强投资拉动能力和发展后劲。

全力以赴促投资。围绕工业升级、陆港建设、环湖开发、农业产业化，实施好58个重点产业项目，完成投资163亿元，力促产业结构不断优化。围绕城乡一体发展，加快环境治理、基础设施等40个关系民生发展的重点项目建设，完成投资76亿元，推动区域协调发展。创新项目服务方式，提高项目服务水平，以大项目带动大投资，推动大发展，确保全社会固定资产投资达到548亿元。

千方百计争项目。紧盯国家、自治区政策和专项资金扶持方向，围绕重点支持领域，精心谋划大项目，充实完善项目库，确保储备项目总额在660亿元以上。加强与国家、自治区有关部门的对接，扎实做好项目前期和申报工作，积极争取列入全国生态文明先行示范区、银川-石嘴山承接产业转移示范区和资源综合利用“双百工程”试点，力争更多项目纳入国家、自治区规划和项目计划。全力对接大唐国际火电、宁夏电投低热值煤发电等项目，争取早日批复。积极争取国家和自治区各类资金支持，全年争取资金同比增长15%。

持之以恒抓招商。把招商引资作为扩大投资的主攻方向，突出招大引强和集群配套，围绕产业链、设备链和资源链，彰显区位、市场等比较优势，积极承接东中部地区产业转移。依托行业协会和专业展会，加强与央企和全国500强企业的联系对接，精心组织重大招商活动，努力引进一批对拉长产业链、促进转型升级、扩张经济总量有重大影响的优质项目，推动北方国际贸易服务中心、中节能环保、中电新能源、西子控股装备、徐工机械制造、明阳集团新能源、雨润农产品加工基地等项目早日落地。规范招商项目认定，严禁职能部门多头切分项目。力争全年引进过亿元项目90个以上，招商实际到位资金同比增长20%以上。

(二)抓改革创新，增强持续发展活力。全面深化改革，充分发挥体制机制改革的牵引作用，以改革创新破解发展难题，以改革创新增强发展活力，推进经济社会转型发展、科学发展。

深化行政体制改革。以加快转变政府职能为目标，完成市、县区政府机构改革，推进事业单位分类改革和法人治理结构建设试点，对市级政府非常设机构进行清理，压减三分之二以上。建立行政审批制度改革工作部门联席会议制度，承接好国家、自治区下放我市的审批事项，继续对市级行政审批事项进行清理下放，最大限度减少行政审批事项。推进行政审批流程标准化、规范化、高效化，推行并联审批、网上审批。加快公共服务中心建设，将部门行政审批事项和相关服务事项全部集中办理。完成公共资源电子交易平台建设。探索整合行政执法权，减少行政执法主体，着力解决权责交叉、多头执法问题。把政府作用与市场力量结合起来，切实把该放的权力放下去、放到位，该管的事情管起来、管到位，充分发挥市场对资源配置的决定性作用，释放市场发展潜力。

推进经济体制改革。做好市属国有企业改革工作，完善现代企业制度，采取合并整合、重组改制、有序退出、扶持壮大等举措，着力打造星瀚、矿业、九柱、善道、文化旅游五大产业集团。组建国有资本运营公司，制定《国有资本经营收益收缴管理办法》，合理确定收益上缴财政比例，不断提高国有资本运营质量和国有资产监管成效。清理废除对非公有制经济的不合理规定，消除各种隐形壁垒。鼓励非公有制企业参与国有企业改制重组，发展混合所有制经济。深化财税体制改革，改进预算编制办法，引入第三方开展项目绩效评价试点，提高财政资金使用效益。清理规范重点支出同财政收支增幅挂钩事项，清理规范市本级各类优惠政策，清理规范各类收费项目。全面梳理定价事项，完善价格调控手段，稳定粮食、蔬菜和肉类价格。认真落实国家结构性减税政策，做好营改增工作。加快完善集约节约用地制度，探索建立工业用地二级储备开发机制和工业用地阶梯地价机制，依法依规清理圈而不建、多圈少建等闲置土地，提高土地利用率。深化农村产权制度改革，在全区率先完成农村土地承包经营权确权登记和农村房屋确权颁证。深入推进平罗县农村土地改革试验区建设，打造全国农村土地经营管理制度改革先行区。

完善农民承包地流转及承包经营权抵押、担保制度，建立农民住房财产权抵押、担保转让制度，探索农民“三权”自愿退出补偿和收储机制，筹建石嘴山市农村产权交易中心，盘活农村资产资源。创新农业经营方式和投资机制，探索建立农民股份合作制度，培育新型农业经营主体。推行农村乡镇治理结构改革试点，激发农村发展活力。加快投融资体制改革，创新融资模式，推进直接融资，争取星瀚集团发行8亿元城投债，西北骏马进入证监会上市审核“绿色通道”，2-3家企业在场外股权交易市场或股权转让系统挂牌上市。强化民间融资监管，切实维护金融秩序。组建担保公司，引进区内外金融机构在我市设立分支机构。

加快社会事业改革。建立促进就业创业长效机制，加强创业基地、培训机构品牌建设。以提高教育质量为重点，深化教育领域改革，实行校长、教师交流轮岗，提高农村学校办学质量，推进教研员竞聘上岗，探索新建公办幼儿园政府购买服务机制。创新职业教育发展模式，深化产教融合、校企合作，完善石嘴山联合学院基础设施配套，进一步扩大职业教育规模，积极培育职业教育产业。创新人才工作机制，加强现有人才的培养和使用，注重重点紧缺人才的引进。加快组建文化产业集团，鼓励非公有制文化企业发展。整合石嘴山日报社、市广播电视台、网络信息中心，推动传统媒体和新兴媒体融合发展。深化医药卫生体制改革，推进公立医院改革，启动全民健康保障信息化工程，健全重特大疾病医疗保险和救助制度。全面实施卫生惠民计划，严格落实10类43项基本公共卫生服务项目，提高卫生服务均等化水平。加快在殡葬、养老服务等行业推行市场化运作，加强行业自律，激发社会组织活力。完善住房公积金归集、提取、使用政策。

(三)抓自身建设，提升服务发展能力。以抓铁有痕的决心推进政府职能转变，深化行政体制和政府机构改革，创新行政管理方式，增强政府的公信力和执行力，切实履行公共服务、市场监管、社会管理、环境保护等职责，努力在建设法治政府和服务型政府上有新作为。

提升依法行政水平。坚决贯彻落实市委决策，严格执行人大及其常委会的决议、决定，积极支持政协履行职能，畅通与人大代表、政协委员沟通联系的绿色通道，认真办理人大代表建议和政协委员提案。加强法治政府建设，严格依照法定权限和程序履行职责、行使权力，主动接受社会各界及新闻媒体监督，积极构筑网络问政平台。健全重大问题集体决策制度，认真执行重大事项专家咨询、社会稳定风险评估、公示听证和合法性审查制度，建立决策责任追究制度，确保各项决策和行政行为依法依规，更加符合发展实际，更加符合人民群众的愿望。

提高行政服务效能。加强电子政务建设，实现网上办公和网上办事，提高政务信息化水平。开展“电视问政”，建设“政务微博”，畅通社情民意。切实将会议活动、文件简报、节会论坛、考核评比和表彰活动的数量减下来，提高工作实效。建立健全抓落实的领导责任制和工作机制，强化督查督办和绩效考评，确保各项工作快节奏、高质量完成。将创新作为提升政府效能的前提和动力，以创新的思维谋划工作，以创新的举措推进落实，各项工作都争创一流水平，努力在全区争先进位，带动政府整体工作上水平、上台阶。

强化勤政廉政建设。严格贯彻落实《党政机关厉行节约反对浪费条例》和“八项规定”，牢固树立过紧日子的思想，提倡勤俭节约，反对铺张浪费。制定本级公务卡强制结算目录和公务接待管理办法，健全预算公开机制，对本级财政、部门“三公”经费预决算进行全面公开，严格控制“三公”支出。探索建立规范合理的政府债务管理及风险预警机制，防范和化解财政金融风险。推行部门权力清单制度，依法公开权力运行流程。加大行政效能监察和问责力度，坚决治理吃拿卡要报、庸懒散软奢等不良风气，严肃查处不作为、慢作为、乱作为等行为。强化行政监察和审计监督，加大从源头上预防和治理腐败的力度，树立廉洁政府形象。

各位代表!我们面临的任务艰巨而繁重，我们肩负的责任重大而光荣。让我们在市委的坚强领导下，在市人大、政协的监督支持下，团结带领全市人民，锐意进取，扎实工作，为加快建设开放富裕和谐美丽的新型工业城市、在全区率先全面建成小康社会而努力奋斗!
